# Improving hit discovery by integrating activity cliff sensitivity into active learning

**DOI:** 10.1093/bioinformatics/btag302

**Published:** 2026-07-07

**Authors:** Junha Kim, Youngkuk Kim, Bonil Koo, Dongmin Bang, Sun Kim

**Affiliations:** Department of Computer Science and Engineering, Seoul National University, Seoul, 08826, Republic of Korea; Department of Computer Science and Engineering, Seoul National University, Seoul, 08826, Republic of Korea; AIGENDRUG Co., Ltd, Seoul, 08758, Republic of Korea; Interdisciplinary Program in Bioinformatics, Seoul National University, Seoul, 08826, Republic of Korea; Interdisciplinary Program in Bioinformatics, Seoul National University, Seoul, 08826, Republic of Korea; Department of Computer Science and Engineering, Seoul National University, Seoul, 08826, Republic of Korea; AIGENDRUG Co., Ltd, Seoul, 08758, Republic of Korea; Interdisciplinary Program in Bioinformatics, Seoul National University, Seoul, 08826, Republic of Korea; Interdisciplinary Program in Artificial Intelligence, Seoul National University, Seoul, 08826, Republic of Korea

## Abstract

**Motivation:**

Active learning has emerged as an effective strategy for accelerating molecular discovery under limited labeling budgets. However, existing methods primarily focus on global information, often overlooking activity cliff—sharp changes in bioactivity caused by small structural perturbations—leading to suboptimal sample selection. In this work, we propose a model-agnostic, activity cliff–aware active learning framework designed to improve hit discovery efficiency without imposing constraints on the underlying molecular representations. Our framework introduces an auxiliary activity cliff scoring module trained on pairwise molecular relationships to explicitly capture local structure–activity sensitivity. The outputs of this module are integrated into a cliff-aware acquisition function that prioritizes structurally informative molecules whose labels are expected to be most beneficial for model improvement. Notably, the proposed strategy is agnostic to backbone architectures and molecular feature, enabling seamless integration with a wide range of existing active learning pipelines.

**Result:**

We evaluate our approach on multiple benchmark datasets under a fixed labeling budget. Across all targets, the proposed method consistently identifies more active compounds than baseline acquisition strategies, demonstrating improved robustness in early-stage, data-scarce learning scenarios. Ablation studies further confirm the contribution of activity cliff awareness to the observed performance gains. Overall, our results underscore the importance of explicitly modeling activity cliffs within active learning frameworks and highlight the effectiveness of a model-agnostic design for accelerating hit discovery in data-limited drug discovery settings.

**Availability and implementation:**

The source code is accessible online at https://github.com/wnsgk/AC-Active.

## 1 Introduction

**Figure 1:**
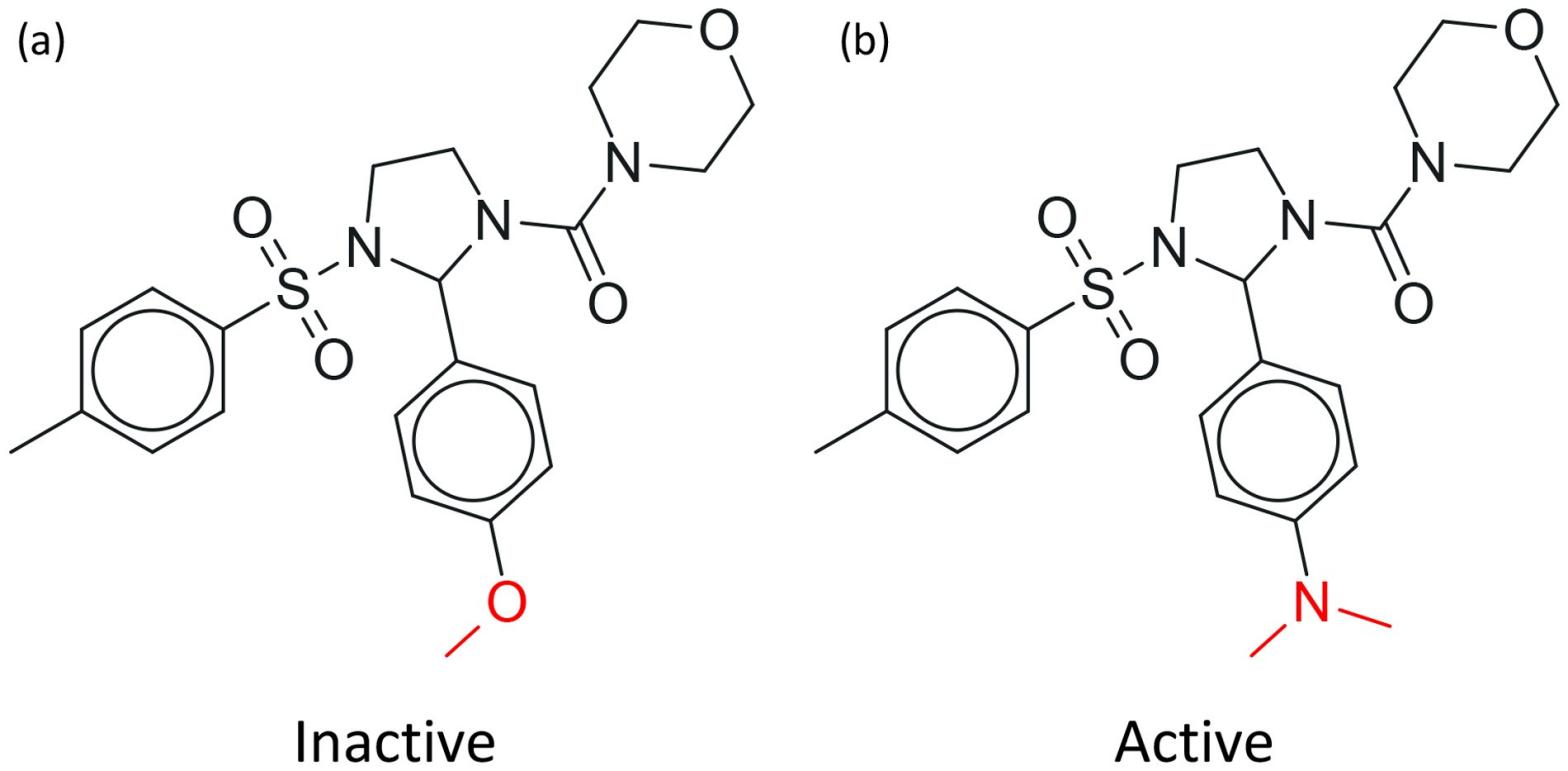
An example of an activity cliff relationship for the ALDH1 target. Molecules (a) and (b) share highly similar chemical structures; however, (a) is inactive while (b) is active against ALDH1.

In the drug discovery process, synthesizing new compounds and experimentally verifying their biological activity require substantial time and financial resources (Paul *et al.* 2010, DiMasi *et al.* 2016). Consequently, identifying promising candidates as early as possible is crucial for improving the overall efficiency of the development pipeline (Hughes *et al.* 2011, Schneider 2018). However, the relationship between molecular structure and biological activity cannot be fully explained by structural similarity alone. Even molecules that share highly similar scaffolds can exhibit dramatically different activities due to subtle variations in functional groups—a phenomenon known as the *activity cliff* (Fig. 1; Maggiora 2006, Stumpfe *et al.* 2019). Such structure–activity discontinuities fundamentally conflict with the assumption of smooth structure–activity relationships that underlies many similarity-based approaches, thereby increasing the risk of missing highly active compounds during early-stage hit identification.

Despite recent advances in computational and machine learning–based methods for hit discovery, activity cliffs remain a major challenge for accurate molecular bioactivity prediction (Gawehn *et al.* 2016, Ouma *et al.* 2024). Most machine learning models rely heavily on structural similarity as a primary signal, which limits their ability to distinguish activity cliff pairs in which structurally similar molecules display markedly different activities (Van Tilborg *et al.* 2022, Deng *et al.* 2023). This limitation is particularly pronounced in the low-data regimes characteristic of early-stage drug discovery, where predictive errors can lead to the exclusion of promising candidates from experimental evaluation. Therefore, developing predictive frameworks that can explicitly account for activity cliff relationships is essential for improving the efficiency and reliability of early hit discovery.

The problem addressed in this study is to efficiently identify hit candidates for a target protein using active learning, given a limited number of initial experimental activity labels and predefined chemical space. In early-stage drug discovery, the amount of experimentally obtainable bioactivity data is extremely limited, and under such low-data conditions, predictive models often struggle to accurately estimate molecular activity (Walters and Murcko 2020, Van Tilborg and Grisoni 2024). Therefore, it is necessary to predict the bioactivity of each compound from limited experimental data and to determine the optimal next compounds to assay using selection criteria considering activity cliffs, in order to effectively explore the chemical space and identify active candidates.

To enable efficient hit candidate discovery under limited labeling conditions, we propose *ACActive*, an activity-cliff-aware active learning framework that can be integrated with arbitrary backbone models. ACActive is designed as a model-agnostic framework, allowing seamless integration with a wide range of backbone architectures, including Graph Neural Network (GNN) based models and fingerprint-based molecular encoders.

ACActive quantifies the degree to which pairs of structurally similar compounds exhibit activity cliff relationships. It explicitly learns activity cliff relationships by sampling structurally similar molecular pairs from the labeled set. It trains an activity cliff scoring module that operates on molecular representations produced by the backbone model to predict whether a given pair forms an activity cliff. This module captures subtle structural variations that lead to significant bioactivity differences without imposing constraints on the base model architecture.

By jointly considering bioactivity predictions and the learned activity cliff scores within a unified acquisition function, the proposed strategy enables more effective exploration of the chemical space and guides the selection of compounds for subsequent experimental labeling.

Extensive experiments on benchmark datasets demonstrate that ACActive consistently outperforms existing active learning methods in early-stage compound screening, highlighting its effectiveness for hit candidate discovery under low-data conditions. By leveraging activity cliff-aware structural information, our framework mitigates structure–activity discontinuities arising from activity cliffs, thereby facilitating effective hit discovery.

## 2 Related work

### 2.1 Active learning for molecular discovery

Active learning has emerged as a powerful paradigm for accelerating molecular discovery, particularly in low data regimes where labeled samples are scarce (Graff *et al.* 2021, Cao *et al.* 2024). While active learning may be susceptible to sampling bias and depends on the quality of initial data, it overcomes the prohibitive costs and time constraints of traditional high-throughput screening (HTS) by strategically prioritizing the most informative molecules. Bioactivity labels are typically obtained from wet-lab assays that measure the interaction between molecules and biological targets. These experiments are time-consuming and costly to acquire, requiring days to weeks per compound. By iteratively selecting informative samples for labeling, active learning effectively reduce the experimental cost associated with high-throughput screening. Previous works include uncertainty-based selection, diversity-based selection, and considering both (Sener and Savarese 2018, Schneider *et al.* 2022, Subedi *et al.* 2024). In addition, reinforcement learning has been explored to learn molecular selection policies in active learning settings (Chen *et al.* 2025). Recent work demonstrated that active deep learning can efficiently traverse chemical space and yields large gains in finding active molecules under low data contraints (Dodds *et al.* 2024, Van Tilborg and Grisoni 2024, Deng *et al.* 2025). Despite these advances, most existing approaches rarely consider chemically meaningful phenomena such as activity cliff. Addressing these discontinuities is essential for constructing models that can guide more chemically informed selection in iterative discovery cycles.

### 2.2 Activity cliffs in drug discovery

The Activity Cliff phenomenon, where minor structural changes lead to drastic activity shifts, has been recognized as a challenge for Quantitative Structure Activity Relationship (QSAR) and machine learning for drug discovery (Maggiora 2006, Stumpfe *et al.* 2019). ACs violate the underlying continuity assumption of most QSAR models, causing them to fail at these critical decision boundaries.

Recent studies have approached this problem by leveraging metric learning such as contrastive learning and triplet loss to increase sensitivity to subtle structural variations (Shen *et al.* 2024, Yu *et al.* 2025). Other work had treated cliff molecules as hard samples and adopted curriculum learning strategies that progressively adapt the model to these challenging cases (Yang *et al.* 2025). Hu *et al.* (2025) have proposed a reinforcement learning framework for de novo drug design that leverages predefined activity cliff scores to guide molecule generation toward structurally sensitive regions of chemical space.

In our work, we extend these ideas by introducing an activity cliff scoring module trained jointly with a multi-view molecular backbone. This module not only enhances the representation’s ability to distinguish cliff-informing molecular pairs but also informs the acquisition stage of active learning, enabling the selection of cliff-sensitive samples in subsequent iterations.

## 3 Materials and methods

### 3.1 Framework overview

**Figure 2:**
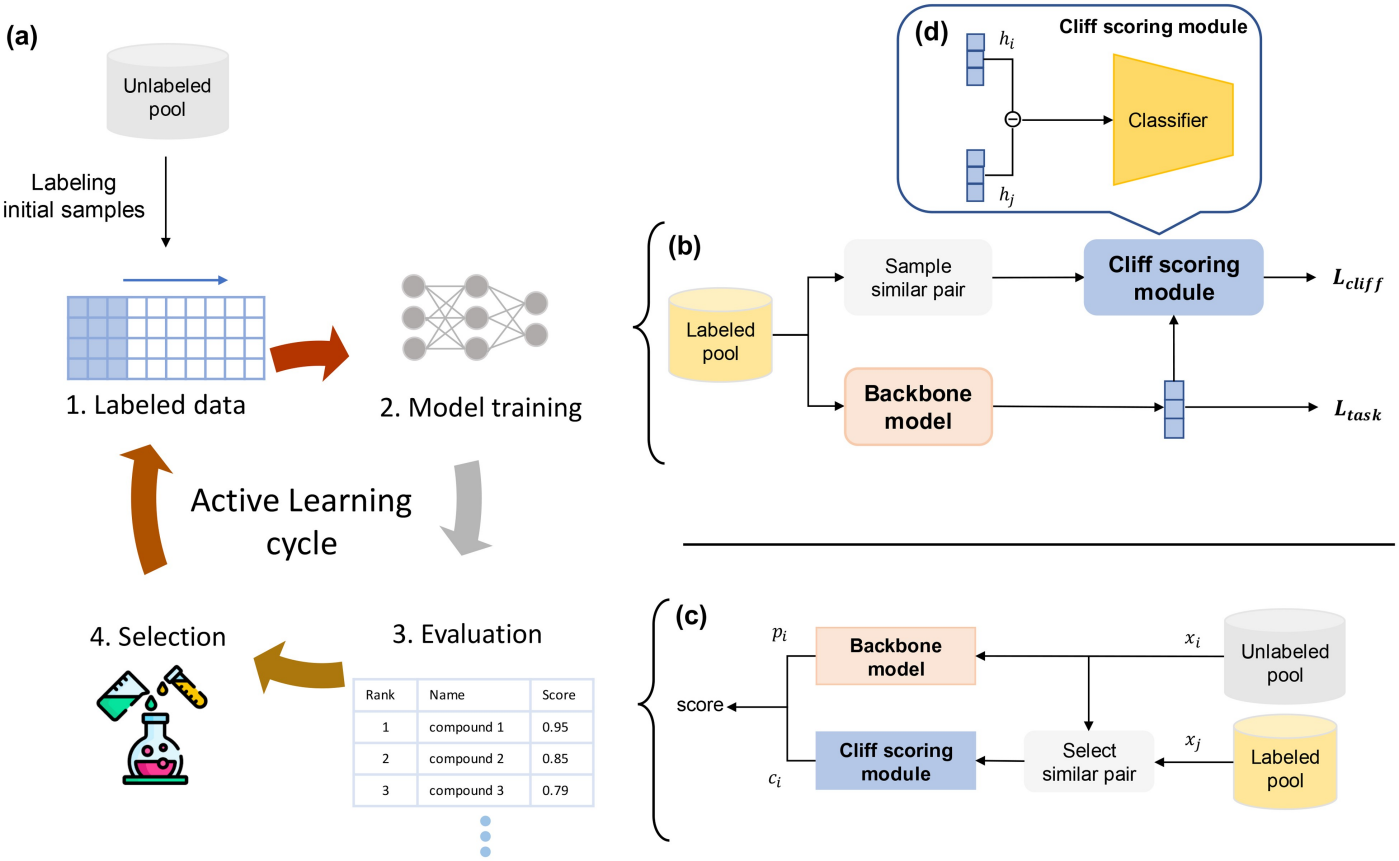
Overall architecture of ACActive and the active learning framework. (a) The active learning loop iteratively expands the labeled set by selecting informative molecules from the unlabeled pool. (b) The model training stage learns a molecular representation and optimizes both the backbone predictor and the activity cliff scoring module. (c) After evaluation, unlabeled molecules are ranked based on the predicted scores to guide the selection process. (d) The cliff scoring module explicitly models activity cliff relationships between structurally similar molecules.

This section provides an overview of the proposed activity cliff aware active learning framework and active learning senarios in this work (Fig. 2). The overview of ACActive, including the training and selection stages, is summarized in Algorithm 1.

Let $X=\{x_{1},x_{2},\ldots ,x_{N}\}$ denote a predefined chemical space for a target protein, where each molecule $x_{k}\in X$ is associated with an unknown biological activity $y(x_{k})\in [0,1]$. The objective of this work is to efficiently identify hit candidates with high activity under a limited experimental labeling budget using active learning.

At iteration *t*, the framework maintains a labeled set $D_{t}=\{(x_{k},y(x_{k}))\}$ and an unlabeled pool $U_{t}=X\setminus D_{t}$. A molecular representation backbone is used to learn a bioactivity predictor $f_{\theta }:X\to R$, which estimates the activity of individual compounds. In addition, we introduce an auxiliary *activity cliff scoring module* $g_{\phi }$ that assigns a cliff score $s(x_{i},x_{j})$ to a pair of molecules, reflecting the degree to which the pair exhibits activity cliff–like behavior, i.e. large activity differences despite high structural similarity. Importantly, this scoring module operates independently of the backbone architecture and can be applied to any molecular encoder.

Based on the predictions from $f_{\theta }$ and the cliff scores from $g_{\phi }$, a cliff-aware acquisition function a(x) is defined over the unlabeled pool $U_{t}$ to select a batch of compounds for experimental evaluation. The selected molecules are then assayed to obtain their true activities and added to the labeled set, yielding $D_{t+1}$. This iterative process continues until the labeling budget is exhausted, enabling efficient exploration of the chemical space while explicitly accounting for structure–activity discontinuities.

### 3.2 Activity cliff scoring module

To incorporate information about structure-activity discontinuities into the active learning process, we introduce an activity cliff scoring module trained jointly with the main bioactivity classifier. This auxiliary module operates on pairs of structurally similar molecules and predicts the likelihood that they form an activity cliff—that is, whether two molecules with similar structures exhibit different bioactivity outcomes. The module provides complementary information for molecule selection in the subsequent active learning round, helping the framework prioritize compounds that are both informative and potentially active.

#### 3.2.1 Activity cliff pair construction

During training, molecule pairs $\left(x_{i},x_{j}\right)$ are sampled from the labeled dataset based on their structural similarity (Fig. 2b). We compute the Tanimoto similarity $S_{ij}$ between molecular fingerprints and select pairs with high similarity ($S_{ij}\geq 0.6$) to focus on structurally similar molecule pairs. Following prior studies, we set the threshold on the Tanimoto similarity to 0.6 (Van Tilborg *et al.* 2022, Yang *et al.* 2025). For each pair, a cliff label is assigned according to the difference between their bioactivity labels:


(1) $$y_{ij}^{\text{cliff}}=\{\begin{array}{cc} 1, & \text{if}\;|A_{i}-A_{j}|\geq \delta ,\\ 0, & \text{otherwise}\; \end{array}$$

where $A_{i}$ and $A_{j}$ denote the binary activity labels of molecules $x_{i}$ and $x_{j}$, respectively and δ is threshold and set to 1 in classification task. A pair is thus considered an activity cliff when two structurally similar compounds exhibit different bioactivity outcomes—that is, one is active while the other is inactive. This formulation captures discontinuities in the structure–activity landscape while remaining consistent with binary activity prediction tasks.

#### 3.2.2 Module architecture

Each molecule pair is first encoded by backbone model, producing molecular representations $h_{i}$ and $h_{j}$. The module computes a differential representation by taking the element-wise difference between the two embeddings, enabling the model to capture differences between the two molecular representations (Fig. 2d). The absolute value is applied to enable permutation invariance of the pairwise representation,


(2) $$h_{\text{diff}}=|h_{i}-h_{j}|.$$

This vector is passed through a lightweight multilayer perceptron (MLP) followed by a sigmoid output to estimate the probability that the pair forms an activity cliff,


(3) $$p_{ij}^{\text{cliff}}=g_{\phi }(h_{\text{diff}}).$$

The module is trained using a binary cross-entropy loss $L_{\text{cliff}}$, optimized jointly with the main bioactivity prediction objective $L_{\text{cls}}$


(4) $$L=L_{\text{cls}}+\lambda \cdot L_{\text{cliff}}.$$

### 3.3 Sample selection

To determine which compounds should be queried in the next active learning round, we design a cliff-aware acquisition function that evaluates each unlabeled molecule based on both its predicted activity and its sensitivity to local structural changes (Fig. 2c). By incorporating these complementary signals, the function prioritizes candidates that are not only likely to be active but also informative about activity cliffs, thereby yielding greater improvements in model performance.

**Algorithm 1** Overview of ACActive Framework **Require**: Labeled dataset $D_{0}$, unlabeled pool $U_{0}$, rounds *T*, budget *B*

**Require**: Backbone model $f_{\theta }$, classifier c(·),cliff scoring module $g_{\phi }(\cdot )$

 1:  **for**t=0**to**T−1**do** 

 2:   Train $f_{\theta },c,g_{\phi }$ on $D_{t}$ by minimizing joint loss L   where $L=L_{\text{cls}}+\lambda L_{\text{cliff}}$

 3:  Construct similar pairs $\left(x_{i},x_{j}\right)$ with   Tanimoto similarity

 4:   Update $g_{\phi }$ to predict $\mathrm{Pr}(y_{ij}^{\text{cliff}}|x_{i},x_{j})$

 5:   **for** each $x_{i}\in U_{t}$**do** 

 6:    $h_{i}\leftarrow f_{\theta }(x_{i})$,  $p_{u}\leftarrow c(h_{i})$

 7:    $N_{i}\leftarrow \text{sim}(x_{i},x_{j})\geq 0.6,\;x_{j}\in D_{t}$

 8:    $p_{ij}^{\text{cliff}}\leftarrow g_{\phi }(h_{i},h_{j})$

 9:    $c_{i}\leftarrow A\text{GGREGATE}{\left\{p_{ij}^{\text{cliff}}\right\}}_{x_{j}\in N_{i}})$

 10:     $s_{i}\leftarrow p_{i}+\alpha c_{i}$

 11:     **end for** 

 12:     $Q_{t}\leftarrow T\text{OPN}(U_{t},\{s_{i}\})$

 13:     Query labels for $Q_{t}$ and update $D_{t+1},U_{t+1}$

 14:  **end for** 

For each unlabeled molecule $x_{i}$, we first obtain its predicted activity probability from the bioactivity classifier:

(5) $$p_{i}=f_{\theta }(x_{i}).$$

Next, we identify labeled neighbors of $x_{i}$ based on structural similarity between two molecules. Specifically, we collect molecules $x_{j}$ from the labeled pool such that their Tanimoto similarity with $x_{i}$ satisfies $S_{ij}\geq 0.6$, forming the neighbor set $N_{i}$. For each neighbor $x_{j}\in N_{i}$, the pair $\left(x_{i},x_{j}\right)$ is passed through the activity cliff scoring module to compute the cliff probability:


(6) $$p_{ij}^{\text{cliff}}=g_{\phi }(x_{i},x_{j}).$$

The cliff probability provides directional corrections conditioned on the known label $A_{j}$ of $x_{j}$: a high $p_{ij}^{\text{cliff}}$ suggests that $x_{i}$ is likely to have the opposite label to $x_{j}$. We aggregate these pairwise signals into a cliff score $c_{i}$:


(7) $$c_{i}=\frac{1}{\left|N_{i}\right|}\sum_{x_{j}\in N_{i}}{\left(-1\right)}^{A_{j}}p_{ij}^{\text{cliff}},$$

where $A_{j}\in \{0,1\}$ is the known activity label of molecule $x_{j}$. Here, ${\left(-1\right)}^{A_{j}}$ assigns a positive sign when the neighbor is inactive and a negative sign when the neighbor is active.

Finally, the acquisition score is computed as:


(8) $$s_{i}=p_{i}+\alpha \cdot c_{i}.$$

Molecules in unlabeled set are ranked by acquisition score $s_{i}$, and the top ranked molecules are added to the labeled set for the next iteration training.

### 3.4 Experiments

#### 3.4.1 Datasets

To evaluate the proposed model, we used two benchmark datasets, LIT-PCBA (Tran-Nguyen *et al.* 2020) and Enamine (Enamine Ltd 2025). The LIT-PCBA benchmark is a curated collection of bioactivity prediction tasks derived from experimentally measured data in PubChem BioAssay. Each dataset contains small molecules represented by canonical SMILES along with binary activity labels. Consistent with previous studies (Van Tilborg and Grisoni 2024, Chen *et al.* 2025), we used three protein targets, ALDH1, PKM2, and VDR and the 100 000 molecules sampled for each target and conducted all experiments on these fixed subsets (Table 1). Enamine is a large-scale library of purchasable chemical compounds used for virtual screening. We used two subsets of Enamine, namely Enamine50k and EnamineHTS, which contain activity values for thymidylate kinase (Graff *et al.* 2021). Following (Chen *et al.* 2025), Enamine50k consists of 50 240 molecules, including 500 active molecules. EnamineHTS consists of 2.1 million molecules, including 1000 active molecules.

**Table 1:** Screening library size and average number of hits in the starting set for each dataset.

Dataset	Screening library size	Hit ratio
ALDH1	100 000	4986 hits (5.0%)
PKM2	100 000	223 hits (0.2%)
VDR	100 000	239 hits (0.2%)
Enamine50k	50 240	500 hits (1.0%)
EnamineHTS	2 141 514	1000 hits (0.05%)

#### 3.4.2 Experimental setup

As shown in Fig. 2a, we begin the active learning process with an initial labeled set for each protein target. These initial samples were uniformly selected from the full dataset while ensuring that at least one active compound is included. At each iteration, the model is retrained from the scratch with the currently labeled set, and a fixed number of new samples are selected from the unlabeled pool using the proposed acquisition function. This iterative selection process is repeated until the predefined labeling budget for each dataset is exhausted.

For the LIT-PCBA benchmarks, we initialize the labeled set with 64 molecules and acquire 64 molecules per iteration, resulting in a total labeling budget of 1000 samples. For Enamine50k, the active learning process is initialized with 500 labeled molecules (1% of the dataset), and the total labeling budget is set to 3000 molecules. For EnamineHTS, we consider two experimental settings to evaluate performance under different sampling budgets. In the first setting, the labeled set is initialized with 2100 molecules (0.1% of the dataset), and the total labeling budget is 12 600 molecules (0.6%). In the second setting, we initialize with 4200 molecules (0.2%) and allow a total budget of 25 200 molecules (1.2%) (Graff *et al.* 2021, Chen *et al.* 2025).

We evaluated the effectiveness of our model and each acquisition strategy by enrichment factor (EF), which quantifies how many more active compounds are discovered compared to random selection under the limited sample labeling budget (Van Tilborg and Grisoni 2024). A higher enrichment factor indicates that the method prioritizes informative and biologically relevant molecules more efficiently.


(9) $$\text{EF}=\frac{N_{\text{model}}}{N_{\text{random}}}.$$

Here, $N_{\text{model}}$ denotes the number of active compounds identified by the model under a fixed labeling budget, and $N_{\text{random}}$ denotes the expected number of active compounds identified by random selection with the same budget. Also we use retrieval rate (RR) for evaluating Enamine (Graff *et al.* 2021, Chen *et al.* 2025), which measures the fraction of top-N highly active compounds in the entire dataset that are successfully retrieved by the selection strategy.

For performance evaluation, we compare our method against existing active learning approaches, including TcsAL (Van Tilborg and Grisoni 2024), PtAL (Cao *et al.* 2024), and GLARE (Chen *et al.* 2025). Each baseline employs a specific acquisition function to guide sample selection: greedy selection, mutual information (MI), upper confidence bound (UCB), and GLARE (Chen *et al.* 2025). To further demonstrate that the proposed method is model-agnostic rather than tailored to a specific representation, we conducted experiments using multiple backbone models, including a Multilayer Perceptron (MLP), a GNN, and a pretrained GNN model, GraphMVP (Liu *et al.* 2022). All experiments are repeated with five random seeds, and the reported results correspond to the mean performance.

## 4 Results and discussion

### 4.1 Performance comparison on benchmarks

We evaluate ACActive across three target in LIT-PCBA and Enamine, under a limited labeling budget, using enrichment factor as the evaluation metric. As shown in Tables 2 and 3, ACActive consistently outperforms baseline acquisition strategies across all targets, demonstrating robust performance in low-budget screening scenarios. ACActive achieves enrichment factor of 6.303, 7.271, 8.407 on ALDH1, PKM2, and VDR, respectively, with GraphMVP. Especially on ALDH1, ACActive find 10% more active compounds compared to the other baseline. Detailed results can be found in the Supplementary Table. While ACActive may exhibit fluctuations in performance during intermediate acquisition rounds, it ultimately identifies a larger number of active compounds than other baseline selection strategies. Although some baselines, such as GLARE, outperform ACActive at earlier stages for certain targets (e.g. PKM2 around round 10), their discovery rate tends to diminish in later rounds. In contrast, ACActive continues to identify new active compounds across acquisition rounds, resulting in better cumulative performance in the final round. While GLARE primarily optimizes global chemical space exploration, ACActive utilizes a specialized module trained on Activity Cliff information to identify active compounds even in regions with significant bioactivity shifts.

**Table 2:** Performance comparison of acquisition strategies on ALDH1, PKM2, and VDR.

		ALDH1		PKM2		VDR	
Model	Strategy	Round 10	Round 16	Round 10	Round 16	Round 10	Round 16
**MLP**	+greedy	**5.552**	5.307	5.208	5.531	**8.804**	8.288
	+MI	4.803	5.366	5.291	5.655	4.125	5.743
	+GLARE	4.992	5.283	**6.366**	5.779	5.870	7.696
	+ACActive	5.339	**5.677**	5.704	**6.090**	6.583	**8.525**
**GNN**	+greedy	**5.473**	5.689	5.208	4.847	3.014	4.203
	+MI	2.027	2.535	0.744	0.621	1.190	1.066
	+GLARE	5.217	5.252	**6.283**	**6.339**	6.266	7.459
	+ACActive	5.436	**5.960**	5.456	**6.339**	**6.346**	**8.229**
**GraphMVP**	+greedy	5.253	5.905	3.968	3.791	4.601	5.624
	+MI	1.930	2.346	0.744	0.808	1.190	1.125
	+GLARE	5.515	5.779	6.696	7.084	6.266	7.696
	+ACActive	**5.844**	**6.303**	**6.779**	**7.271**	**6.901**	**8.407**

Enrichment factor measured at rounds 10 and 16 for each dataset. Higher values indicate better performance. Bold values indicate the best performance, and underlined values indicate the second-best performance.

**Table 3:** Performance comparison of acquisition strategies on Enamine.

		Enamine50k		EnamineHTS0.1		EnamineHTS0.2	
Model	Strategy	Round 4	Round 6	Round 4	Round 6	Round 4	Round 6
**MLP**	+greedy	0.255	0.433	0.184	0.378	0.205	0.352
	+UCB	0.248	0.411	0.159	0.344	0.219	0.362
	+GLARE	0.235	0.412	0.174	0.377	0.256	0.469
	+ACActive	**0.274**	**0.450**	**0.190**	**0.381**	**0.264**	**0.484**
**GNN**	+greedy	0.537	0.730	0.505	0.735	0.609	0.848
	+UCB	0.555	0.734	0.500	0.715	0.714	0.869
	+GLARE	**0.558**	**0.746**	**0.542**	0.734	0.697	0.868
	+ACActive	0.545	**0.746**	0.511	**0.760**	**0.722**	**0.880**
**GraphMVP**	+greedy	0.580	0.752	0.488	0.728	0.606	0.835
	+UCB	0.576	0.757	0.474	0.720	0.696	0.876
	+GLARE	0.576	0.746	0.506	0.732	0.712	0.862
	+ACActive	**0.581**	**0.760**	**0.521**	**0.779**	**0.718**	**0.888**

Retrieval rate measured at rounds 4 and 6 for each dataset. Higher values indicate better performance. Bold values indicate the best performance, and underlined values indicate the second-best performance.

Importantly, the performance improvements of ACActive are not limited to a specific model architecture (Tables 2 and 3). We observed consistent improvements when ACActive is combined with diverse backbone models, including a simple MLP, a graph neural network (GNN), and a GNN based pretrained model (GraphMVP). This model-agnostic behavior suggests that the proposed activity-cliff–aware acquisition strategy leverages signals that generalize across different molecular representations. By decoupling backbone-based exploitation from activity cliff identification, ACActive provides a general mechanism for prioritizing chemically informative compounds. As a result, the method remains effective across both shallow and deep models, as well as across pretrained and non-pretrained settings, highlighting its broad applicability to different active learning pipelines.

Notably, the advantage of ACActive becomes more pronounced in targets with extremely low active rates such as EnamineHTS.

### 4.2 Ablation study on acquisition function

To analyze the contribution of the proposed acquisition function, we conducted an ablation study that focuses on the role of the cliff score during the selection process (Table 4). In this setting, the activity cliff scoring module is fully retained and trained jointly with the backbone model, allowing the model to learn and leverage activity cliff–related signals during training. However, during the acquisition step, the proposed cliff-based acquisition score is not applied, and candidate molecules are selected solely based on the prediction scores produced by the backbone model.

**Table 4:** Ablation study on ACActive framework.

		ALDH1		PKM2		VDR	
Model	Strategy	Round 10	Round 16	Round 10	Round 16	Round 10	Round 16
**MLP**	+ACActive w/o cliff score	5.168	5.492	5.126	5.966	5.870	7.341
	+ACActive	**5.339**	**5.677**	**5.704**	**6.090**	**6.583**	**8.525**
**GNN**	+ACActive w/o cliff score	5.174	5.634	2.067	6.214	**6.425**	7.223
	+ACActive	**5.436**	**5.960**	**5.456**	**6.339**	6.346	**8.229**
**GraphMVP**	+ACActive w/o cliff score	5.059	5.512	6.366	6.836	5.870	7.815
	+ACActive	**5.844**	**6.303**	**6.779**	**7.271**	**6.901**	**8.407**

Enrichment factor measured at rounds 10 and 16 for each dataset. Higher values indicate better performance. Bold values indicate the best performance.

As shown in Table 4, the original ACActive consistently outperforms the ablated variant that excludes the cliff score from the acquisition process. This performance gap indicates that simply learning activity cliff representations during training is insufficient to achieve optimal performance. Instead, explicitly incorporating the cliff score into the acquisition function plays an important role in guiding the selection toward more informative compounds. These results show that the performance improvements of ACActive is not only from the auxiliary activity cliff prediction task, but more importantly from its explicit use in the selection strategy. Also, we conducted an ablation that removes cliff-aware training while retaining the cliff-based acquisition. Detailed results are provided in Supplementary Section 1.7.

### 4.3 Visualization of selected active compounds

**Figure 3:**
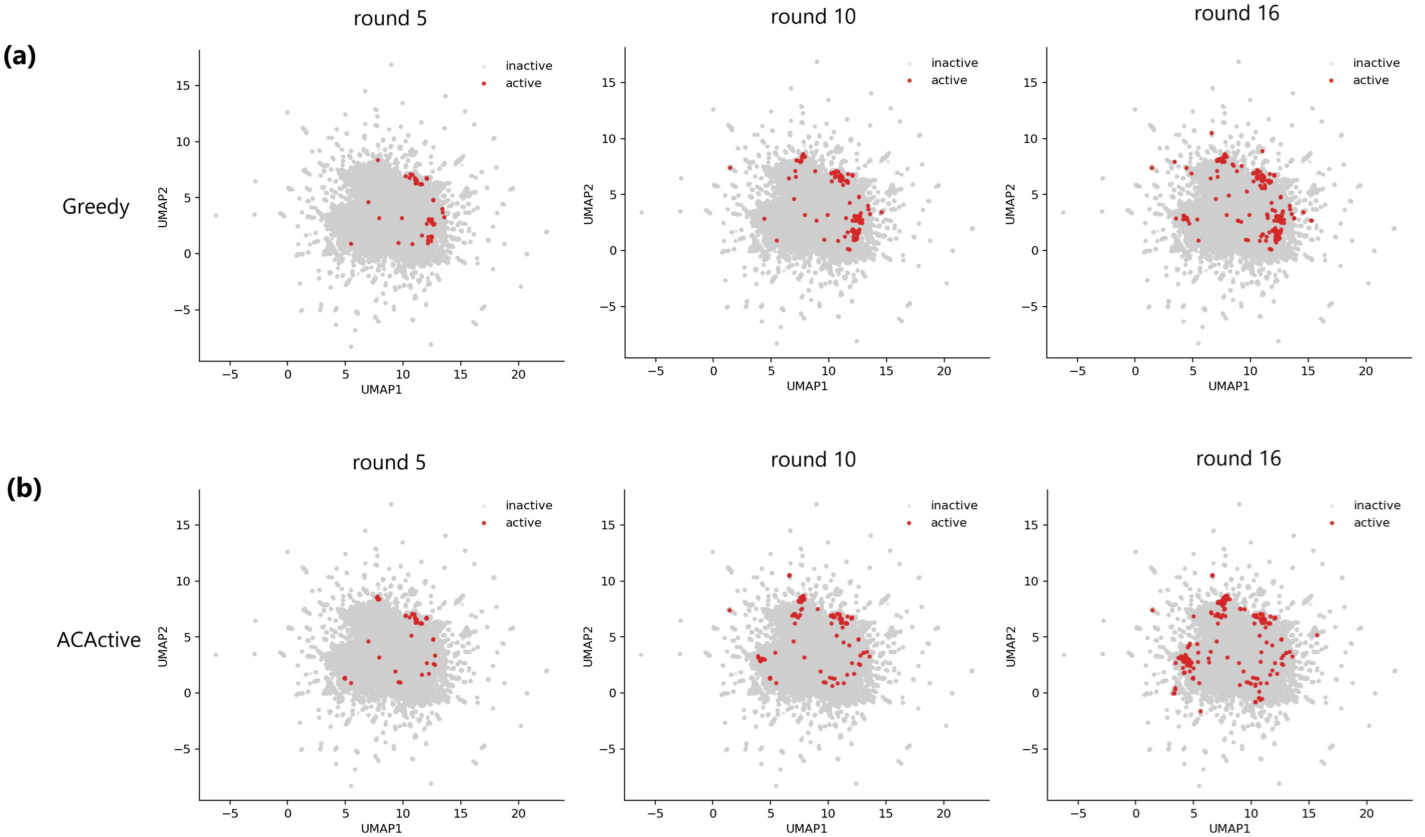
Visualization of selected active compounds. This figure is visualization of selected active compounds on ALDH1 target. (a) Active compounds selected using greedy selection, and (b) active compounds selected using ACActive. From left to right, each panel visualizes the active compounds identified at rounds 5, 10, and 16, respectively.

To further analyze the selection behavior of different acquisition strategies, we visualize the molecular fingerprints of active compounds identified at rounds 5, 10, and 16 in target ALDH1 (Fig. 3). We compare greedy selection and ACActive using GraphMVP as the backbone model. As the acquisition progresses, the greedy selection strategy increasingly concentrates on active compounds located in similar regions of the fingerprint space, indicating repeated exploitation of a narrow chemical neighborhood. In contrast, ACActive progressively identifies active compounds distributed across more diverse regions of the chemical space. From round 5 to round 16, the coverage of the fingerprint space continuously expands under ACActive, suggesting that the proposed method promotes broader exploration by uncovering active compounds from previously unexplored regions rather than repeatedly selecting similar ones.

In contrast to greedy selection, which repeatedly exploits high-confidence predictions within known active regions, ACActive captures uncertainty arising from structure–activity discontinuities around active compounds. This localized uncertainty encourages the selection of compounds near the boundaries of active regions, enabling the discovery of new active neighborhoods and leading to the progressive expansion observed in the fingerprint visualizations.

### 4.4 Case study on selected samples

**Figure 4:**
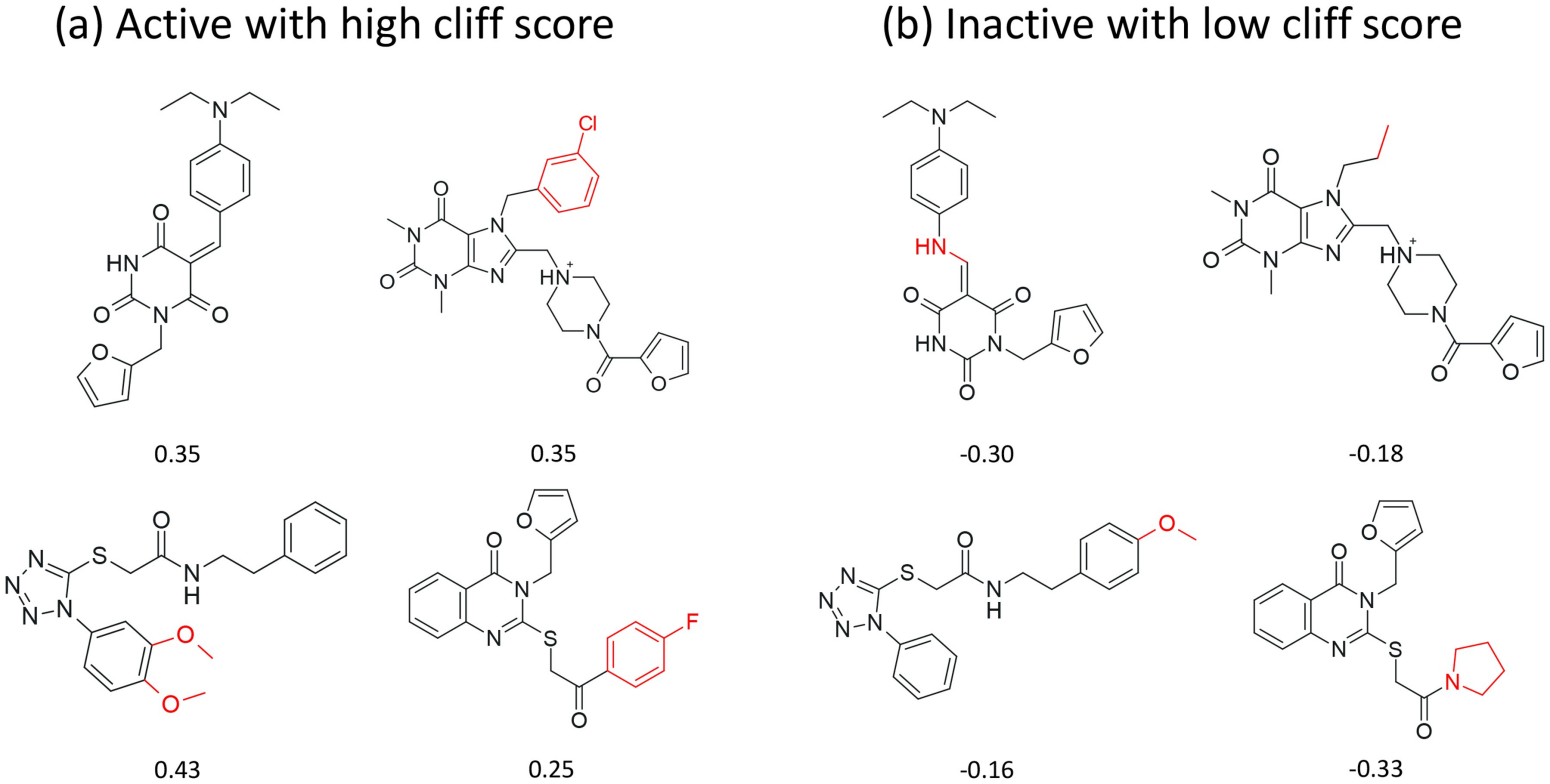
Case study on cliff score. (a) and (b) depict compound pairs that form activity cliff relationships. The cliff score is attatched below. (a) Active compounds assigned high activity cliff scores, which are selected by the proposed acquisition strategy. (b) Inactive compounds involved in activity cliff relationships but assigned low activity cliff scores, and thus not selected. The structural differences between the two molecules are highlighted in red.

We consider a representative pair of compounds with high structural similarity (Tanimoto similarity≥0.6). As shown in Fig. 4a and b, the molecule pair exhibits activity cliff relationships. The backbone classifier assigns nearly identical predicted probabilities to both molecules, yet their cliff scores differ substantially, reflecting the cliff scoring module’s ability to detect subtle local structural variations that may lead to abrupt changes in activity. As a result, the compound with the higher cliff score is prioritized in the acquisition process, reversing the selection order that would arise from probability-based selection alone.

In contrast, probability-based selection, such as greedy, would treat the two compounds as nearly equivalent, failing to account for the potential impact of structure–activity discontinuities on model refinement. This example illustrates how incorporating the cliff score enables the framework to identify structurally informative molecules that conventional active learning strategies would likely overlook.

## 5 Conclusion

In this work, we proposed ACActive, a model-agnostic active learning framework designed to accelerate hit discovery by explicitly leveraging activity cliff information. The proposed approach consistently identified a larger number of active compounds than existing active learning methods, particularly under challenging conditions characterized by limited labeled data and low active rates. By incorporating an activity cliff scoring module, ACActive effectively captures the sensitivity of biological activity to subtle structural variations, enabling more informed and data-efficient molecule selection.

Beyond its empirical performance gains, this study highlights the importance of explicitly modeling activity cliff relationship within active learning pipelines. Our results suggest that activity-cliff–aware modeling is not merely an auxiliary enhancement but a principled strategy for improving data efficiency in molecular discovery. This insight positions activity cliff information as a valuable inductive bias for guiding exploration in low-resource drug discovery settings.

While ACActive demonstrates clear advantages for hit discovery, there remain opportunities for further improvement. Future work may benefit from incorporating richer activity cliff signals derived from large-scale molecular datasets, which could enhance the robustness and generalizability of cliff predictions. Furthermore, the current framework can be extended to continuous activity prediction tasks (e.g. $pIC_{50}$) by redefining activity cliffs based on a regression-based criterion, where structural similarity exceeds a predefined threshold while numerical activity differences surpass a specific margin. In addition, the proposed framework could be naturally extended to closed-loop molecular design settings, for example by incorporating generative models, potentially enabling iterative hit exploration and optimization.

In summary, this study demonstrates that explicitly incorporating activity cliff information substantially improves hit discovery performance in active learning settings. We believe that activity-cliff–aware modeling represents a promising and generalizable direction for advancing data-efficient drug discovery pipelines.

## Supplementary Material

btag302_Supplementary_Data

## Data Availability

All datasets utilized in this study are publicly available. We use LIT-PCBA preprocessed at https://github.com/molML/traversing_chem_space and the Enamine subset modified at https://github.com/biomed-AI/GLARE. The source code is accessible online at https://github.com/wnsgk/AC-Active.
