# Supplementary material for: Improving hit discovery by integrating activity cliff sensitivity into active learning
Source: Bioinformatics. 2026 Jul 7;42(Suppl 1):btag302. doi: 10.1093/bioinformatics/btag302 (PMC13340233; doi:10.1093/bioinformatics/btag302)
Supplement: btag302_Supplementary_Data [file btag302_supplementary_data.pdf]

SUPPLEMENTARY INFORMATION FOR:

Improving Hit Discovery by Integrating Activity Cliff Sensitivity into Active Learning

Junha Kim[1], **Youngkuk Kim**[1,2], **Bonil Koo**[3], **Dongmin Bang**[3], **Sun Kim**[1,2,3,4*]

[1]Department of Computer Science and Engineering, Seoul National University, Seoul 08826, Republic of Korea
[2]AIGENDRUG Co., Ltd., Seoul 08758, Republic of Korea
[3]Interdisciplinary Program in Bioinformatics, Seoul National University, Seoul 08826, Republic of Korea
[4]Interdisciplinary Program in Artificial Intelligence, Seoul National University, Seoul 08826, Republic of Korea

Contents

1 Supplementary Results **2**
1.1 Detailed result on LIT-PCBA . . . 2
1.2 Detailed result on Enamine . . . 6
1.3 Analysis on structural similarity threshold for activity cliff selection . . . 8
1.4 Computational cost . . . 8
1.5 Analysis on hyperparameter sensitivity . . . 9
1.6 Analysis on robustness . . . 10
1.7 Additional ablation study . . . 11
1.8 Statistical analysis . . . 12

2 Supplementary Methods **13**
2.1 Comparison model settings . . . 13
2.1.1 Baseline acquisition strategy . . . 13
2.1.2 Backbone model . . . 13
2.2 Implementation details . . . 13

1 Supplementary Results

1.1 Detailed result on LIT-PCBA

Table S1: Detailed performance of ACActive on ALDH1, PKM2, and VDR

Dataset	ALDH1			PKM2			VDR		
Arch	MLP	GNN	GraphMVP	MLP	GNN	GraphMVP	MLP	GNN	GraphMVP
1	0.966 (0.265)	0.966 (0.265)	0.966 (0.265)	0.877 (0.000)	0.877 (0.000)	0.877 (0.000)	1.043 (0.348)	1.043 (0.348)	1.043 (0.348)
2	2.182 (0.679)	1.991 (0.401)	1.964 (0.589)	1.715 (1.146)	2.183 (1.034)	1.871 (0.795)	1.535 (0.686)	2.149 (2.398)	2.303 (2.703)
3	3.516 (1.074)	3.231 (0.538)	3.212 (0.699)	2.387 (2.246)	2.808 (2.511)	2.246 (1.499)	2.062 (1.150)	2.886 (3.085)	2.749 (2.783)
4	4.011 (1.219)	4.026 (1.005)	3.982 (1.012)	3.574 (3.037)	3.574 (3.368)	2.553 (1.760)	2.488 (1.363)	2.986 (3.254)	3.359 (3.375)
5	4.224 (1.348)	4.603 (1.247)	4.686 (1.355)	3.979 (3.149)	4.213 (3.298)	2.926 (1.923)	3.296 (2.474)	3.523 (3.202)	4.205 (3.383)
6	4.479 (1.523)	4.957 (1.245)	5.136 (1.249)	4.538 (3.768)	4.214 (3.026)	3.458 (2.230)	4.079 (2.325)	4.288 (3.231)	5.543 (3.722)
7	4.527 (1.491)	5.119 (1.298)	5.360 (1.224)	4.717 (4.193)	4.315 (3.382)	5.018 (4.186)	4.456 (1.898)	4.650 (2.834)	5.909 (3.892)
8	4.691 (1.361)	5.092 (1.339)	5.719 (1.249)	4.590 (4.055)	4.403 (3.449)	5.527 (4.204)	5.233 (2.184)	5.413 (2.736)	6.406 (4.168)
9	4.982 (1.325)	5.266 (1.402)	5.798 (1.279)	4.918 (4.057)	4.743 (3.451)	5.972 (4.069)	5.487 (1.980)	5.825 (2.741)	6.585 (4.527)
10	5.339 (1.237)	5.436 (1.347)	5.844 (1.278)	5.704 (4.011)	5.456 (3.482)	6.779 (3.552)	6.583 (1.784)	6.346 (2.999)	6.901 (4.906)
11	5.526 (1.183)	5.610 (1.080)	5.959 (1.232)	6.247 (4.205)	5.622 (3.642)	7.262 (3.709)	7.630 (1.220)	6.508 (2.932)	6.807 (4.763)
12	5.597 (1.052)	5.678 (0.872)	6.091 (1.190)	6.288 (4.005)	5.845 (3.695)	7.176 (3.560)	7.997 (1.528)	7.006 (2.938)	6.935 (4.957)
13	5.647 (0.961)	5.831 (0.739)	6.171 (1.074)	6.115 (3.802)	6.045 (3.524)	7.380 (3.535)	8.058 (1.575)	7.924 (3.375)	7.789 (4.783)
14	5.651 (0.936)	5.888 (0.630)	6.239 (0.840)	6.494 (3.867)	6.092 (3.136)	7.431 (3.681)	8.241 (1.315)	8.305 (3.276)	7.985 (4.827)
15	5.606 (0.828)	5.929 (0.623)	6.204 (0.727)	6.263 (3.647)	6.263 (3.002)	7.413 (3.723)	8.407 (0.955)	8.224 (3.154)	8.163 (4.779)
16	5.677 (0.835)	5.960 (0.673)	6.303 (0.675)	6.090 (3.546)	6.339 (2.838)	7.271 (3.643)	8.525 (0.962)	8.229 (3.084)	8.407 (4.884)

Table S2: Detailed performance of greedy on ALDH1, PKM2, and VDR

Dataset	ALDH1			PKM2			VDR		
Arch	MLP	GNN	GraphMVP	MLP	GNN	GraphMVP	MLP	GNN	GraphMVP
1	0.966 (0.265)	0.966 (0.265)	0.966 (0.265)	0.877 (0.000)	0.877 (0.000)	0.877 (0.000)	1.043 (0.348)	1.043 (0.348)	1.043 (0.348)
2	2.291 (0.860)	1.664 (0.665)	1.719 (0.570)	1.559 (0.986)	1.715 (0.909)	1.715 (0.909)	1.842 (0.614)	1.075 (0.614)	1.228 (0.614)
3	4.011 (1.104)	2.965 (1.237)	3.269 (1.296)	1.544 (1.123)	2.808 (1.830)	2.667 (2.925)	3.161 (1.823)	1.099 (0.825)	1.924 (0.801)
4	4.390 (1.192)	4.084 (1.258)	4.376 (1.259)	2.042 (1.170)	3.830 (3.153)	2.808 (3.090)	3.484 (1.829)	1.493 (1.451)	2.239 (1.154)
5	4.733 (1.440)	4.544 (1.414)	4.697 (1.799)	3.862 (3.364)	4.916 (4.373)	3.511 (4.138)	4.205 (1.419)	2.273 (3.133)	2.841 (1.797)
6	4.927 (1.462)	4.897 (1.588)	4.947 (1.796)	4.971 (4.615)	5.187 (4.630)	3.350 (3.780)	6.276 (1.781)	2.615 (2.883)	3.765 (2.872)
7	5.076 (1.579)	5.128 (1.638)	4.982 (1.784)	5.018 (4.771)	5.218 (4.594)	3.713 (3.682)	7.072 (1.952)	2.616 (2.592)	3.681 (2.980)
8	5.303 (1.579)	5.281 (1.573)	5.062 (1.707)	5.058 (4.907)	5.527 (5.117)	3.934 (3.789)	7.669 (1.997)	2.887 (3.094)	3.699 (2.816)
9	5.448 (1.535)	5.380 (1.336)	5.232 (1.659)	5.094 (4.686)	5.445 (4.791)	4.040 (3.636)	8.611 (1.452)	2.955 (2.985)	3.968 (2.691)
10	5.552 (1.560)	5.473 (1.350)	5.253 (1.624)	5.208 (4.753)	5.208 (4.600)	3.968 (3.609)	8.804 (1.187)	3.014 (2.743)	4.601 (3.179)
11	5.549 (1.452)	5.532 (1.199)	5.426 (1.580)	5.544 (4.371)	5.232 (4.428)	3.748 (3.409)	8.602 (1.183)	3.366 (2.838)	5.236 (3.001)
12	5.515 (1.384)	5.612 (1.073)	5.587 (1.365)	5.697 (4.215)	5.179 (4.198)	3.847 (3.568)	8.563 (1.193)	3.609 (3.055)	5.449 (2.991)
13	5.534 (1.305)	5.619 (1.004)	5.671 (1.255)	5.693 (4.052)	5.131 (3.998)	4.077 (3.690)	8.259 (1.095)	4.029 (3.371)	5.506 (3.140)
14	5.415 (1.284)	5.743 (0.879)	5.748 (1.145)	5.757 (3.912)	5.021 (3.851)	3.950 (3.614)	8.113 (1.022)	4.088 (3.254)	5.558 (3.114)
15	5.339 (1.258)	5.737 (0.782)	5.860 (0.962)	5.624 (3.784)	4.857 (3.629)	3.834 (3.506)	8.285 (1.079)	4.203 (3.350)	5.605 (3.198)
16	5.307 (1.189)	5.689 (0.737)	5.905 (0.891)	5.531 (3.710)	4.847 (3.674)	3.791 (3.383)	8.288 (1.199)	4.203 (3.372)	5.624 (3.221)

Table S3: Detailed performance of MI on ALDH1, PKM2, and VDR

Dataset	ALDH1			PKM2			VDR		
Arch	MLP	GNN	GraphMVP	MLP	GNN	GraphMVP	MLP	GNN	GraphMVP
1	0.966 (0.265)	0.966 (0.265)	0.966 (0.265)	0.877 (0.000)	0.877 (0.000)	0.877 (0.000)	1.043 (0.348)	1.043 (0.348)	1.043 (0.348)
2	2.019 (0.719)	0.791 (0.159)	0.764 (0.067)	1.559 (0.986)	0.936 (0.312)	0.936 (0.312)	1.382 (0.574)	1.075 (0.376)	0.921 (0.307)
3	3.497 (1.033)	0.817 (0.213)	0.988 (0.166)	1.825 (1.140)	0.842 (0.281)	0.983 (0.344)	2.062 (1.567)	1.099 (0.337)	0.962 (0.337)
4	4.128 (1.096)	0.948 (0.627)	1.181 (0.471)	3.574 (2.604)	0.766 (0.255)	0.894 (0.313)	2.364 (1.688)	1.244 (0.556)	0.995 (0.305)
5	4.390 (1.332)	1.195 (1.094)	1.290 (0.623)	3.979 (3.171)	0.702 (0.234)	0.819 (0.287)	2.387 (1.811)	1.364 (0.579)	1.136 (0.359)
6	4.499 (1.361)	1.264 (1.214)	1.563 (0.941)	4.754 (3.976)	0.648 (0.216)	0.864 (0.432)	2.406 (2.001)	1.360 (0.533)	1.046 (0.331)
7	4.690 (1.459)	1.409 (1.340)	1.649 (0.882)	5.118 (4.504)	0.702 (0.401)	0.803 (0.401)	2.616 (1.928)	1.453 (0.306)	1.066 (0.362)
8	4.790 (1.375)	1.579 (1.470)	1.934 (0.774)	5.339 (4.689)	0.656 (0.375)	0.749 (0.375)	2.977 (2.239)	1.353 (0.285)	1.083 (0.361)
9	4.787 (1.338)	1.800 (1.476)	1.921 (0.755)	5.182 (4.609)	0.703 (0.351)	0.703 (0.351)	3.461 (2.552)	1.266 (0.267)	1.013 (0.338)
10	4.803 (1.264)	2.027 (1.583)	1.930 (0.714)	5.291 (4.539)	0.744 (0.309)	0.744 (0.309)	4.125 (2.708)	1.190 (0.251)	1.190 (0.502)
11	4.988 (1.227)	2.186 (1.625)	1.953 (0.682)	5.388 (4.215)	0.781 (0.247)	0.703 (0.292)	4.937 (2.394)	1.122 (0.237)	1.122 (0.473)
12	5.072 (1.068)	2.340 (1.657)	2.013 (0.686)	5.697 (4.056)	0.740 (0.234)	0.666 (0.277)	5.166 (2.188)	1.062 (0.224)	1.132 (0.469)
13	5.214 (1.013)	2.376 (1.739)	2.192 (0.728)	5.974 (4.013)	0.703 (0.222)	0.773 (0.263)	5.439 (2.159)	1.074 (0.251)	1.074 (0.445)
14	5.336 (0.952)	2.420 (1.746)	2.311 (0.846)	5.891 (3.826)	0.669 (0.212)	0.870 (0.268)	5.622 (2.237)	1.022 (0.239)	1.022 (0.424)
15	5.397 (0.974)	2.520 (1.755)	2.348 (0.811)	5.751 (3.732)	0.639 (0.202)	0.831 (0.256)	5.726 (2.291)	1.036 (0.244)	1.036 (0.495)
16	5.366 (0.949)	2.535 (1.817)	2.346 (0.814)	5.655 (3.673)	0.621 (0.197)	0.808 (0.249)	5.743 (2.297)	1.066 (0.237)	1.125 (0.393)

Table S4: Detailed performance of GLARE on ALDH1, PKM2, and VDR

Dataset	ALDH1			PKM2			VDR		
Arch	MLP	GNN	GraphMVP	MLP	GNN	GraphMVP	MLP	GNN	GraphMVP
1	0.966 (0.265)	0.966 (0.265)	0.966 (0.265)	0.877 (0.000)	0.877 (0.000)	0.877 (0.000)	1.043 (0.348)	1.043 (0.348)	1.043 (0.348)
2	1.991 (0.589)	2.237 (0.550)	2.701 (1.203)	1.559 (1.208)	1.715 (0.764)	1.559 (0.697)	1.535 (0.971)	1.228 (0.376)	0.921 (0.307)
3	3.745 (1.010)	3.935 (1.080)	4.410 (1.775)	2.808 (2.082)	2.808 (2.175)	3.088 (2.415)	2.474 (2.443)	2.474 (1.415)	1.787 (1.275)
4	4.303 (1.167)	4.551 (1.628)	5.032 (2.017)	3.191 (2.455)	4.723 (3.885)	4.723 (4.008)	2.737 (2.314)	3.359 (2.380)	1.866 (1.419)
5	4.508 (1.536)	4.650 (1.823)	5.064 (1.778)	4.330 (3.927)	5.501 (4.690)	5.735 (4.943)	3.864 (2.577)	3.637 (2.774)	2.841 (2.215)
6	4.539 (1.699)	4.837 (1.937)	5.265 (1.856)	5.511 (5.015)	5.619 (4.935)	6.267 (4.959)	4.184 (2.958)	4.079 (2.947)	4.184 (2.292)
7	4.733 (1.649)	4.913 (1.920)	5.386 (1.997)	5.720 (5.230)	5.519 (4.577)	6.724 (5.372)	4.844 (2.432)	4.747 (3.485)	4.844 (2.412)
8	4.835 (1.619)	5.016 (1.884)	5.462 (1.976)	6.276 (4.891)	5.808 (4.604)	6.838 (5.050)	5.594 (2.497)	5.143 (3.547)	5.503 (2.757)
9	4.881 (1.603)	5.117 (1.842)	5.475 (1.837)	6.060 (4.559)	6.148 (4.538)	6.939 (4.979)	5.825 (1.818)	5.656 (2.883)	5.825 (2.318)
10	4.992 (1.401)	5.217 (1.751)	5.515 (1.747)	6.366 (4.495)	6.283 (4.432)	6.696 (4.795)	5.870 (1.709)	6.266 (2.706)	6.266 (2.059)
11	5.193 (1.294)	5.399 (1.635)	5.587 (1.756)	6.559 (4.589)	6.481 (4.597)	6.637 (4.743)	6.358 (1.439)	6.433 (2.428)	6.956 (1.047)
12	5.306 (1.276)	5.352 (1.571)	5.638 (1.642)	6.288 (4.402)	6.214 (4.278)	6.732 (4.496)	7.077 (1.433)	6.511 (2.278)	7.218 (0.912)
13	5.308 (1.266)	5.322 (1.546)	5.685 (1.447)	6.115 (4.302)	5.904 (4.065)	6.537 (4.370)	7.319 (1.171)	7.051 (2.825)	7.454 (0.717)
14	5.340 (1.257)	5.274 (1.453)	5.735 (1.367)	5.958 (4.199)	5.891 (3.743)	6.360 (4.148)	7.283 (1.077)	7.219 (2.861)	7.602 (0.998)
15	5.298 (1.239)	5.196 (1.375)	5.725 (1.222)	5.879 (4.164)	5.943 (3.445)	6.518 (3.906)	7.676 (0.448)	7.127 (2.809)	7.554 (1.130)
16	5.283 (1.229)	5.252 (1.331)	5.779 (1.162)	5.779 (3.967)	6.339 (3.292)	7.084 (3.751)	7.696 (0.621)	7.459 (2.683)	7.696 (1.283)

1.2 Detailed result on Enamine

Table S5: Detailed performance of ACActive on Enamine

Dataset	Enamine50k			EnamineHTS0.1			EnamineHTS0.2		
Arch	MLP	GNN	GraphMVP	MLP	GNN	GraphMVP	MLP	GNN	GraphMVP
1	0.011 (0.007)	0.011 (0.007)	0.011 (0.007)	0.002 (0.000)	0.002 (0.000)	0.002 (0.000)	0.003 (0.002)	0.003 (0.002)	0.003 (0.002)
2	0.084 (0.019)	0.160 (0.046)	0.178 (0.055)	0.041 (0.005)	0.086 (0.011)	0.099 (0.008)	0.064 (0.021)	0.170 (0.054)	0.154 (0.033)
3	0.170 (0.031)	0.379 (0.024)	0.402 (0.054)	0.090 (0.014)	0.272 (0.048)	0.297 (0.031)	0.133 (0.033)	0.478 (0.028)	0.493 (0.059)
4	0.274 (0.021)	0.545 (0.026)	0.581 (0.034)	0.190 (0.039)	0.511 (0.043)	0.521 (0.040)	0.264 (0.044)	0.722 (0.013)	0.718 (0.044)
5	0.361 (0.025)	0.670 (0.015)	0.685 (0.032)	0.296 (0.054)	0.663 (0.031)	0.699 (0.015)	0.397 (0.052)	0.822 (0.009)	0.833 (0.014)
6	0.450 (0.022)	0.746 (0.011)	0.760 (0.030)	0.381 (0.055)	0.760 (0.014)	0.779 (0.014)	0.484 (0.051)	0.880 (0.005)	0.888 (0.007)

Table S6: Detailed performance of greedy on Enamine

Dataset	Enamine50k			EnamineHTS0.1			EnamineHTS0.2		
Arch	MLP	GNN	GraphMVP	MLP	GNN	GraphMVP	MLP	GNN	GraphMVP
1	0.011 (0.007)	0.011 (0.007)	0.011 (0.007)	0.002 (0.000)	0.002 (0.000)	0.002 (0.000)	0.003 (0.002)	0.003 (0.002)	0.003 (0.002)
2	0.074 (0.021)	0.163 (0.038)	0.170 (0.051)	0.037 (0.008)	0.056 (0.026)	0.060 (0.027)	0.070 (0.027)	0.039 (0.015)	0.053 (0.026)
3	0.155 (0.031)	0.378 (0.031)	0.408 (0.032)	0.080 (0.008)	0.280 (0.056)	0.303 (0.019)	0.128 (0.048)	0.317 (0.089)	0.280 (0.093)
4	0.255 (0.029)	0.537 (0.011)	0.580 (0.013)	0.184 (0.027)	0.505 (0.045)	0.488 (0.029)	0.205 (0.067)	0.609 (0.075)	0.606 (0.040)
5	0.354 (0.026)	0.649 (0.019)	0.682 (0.014)	0.283 (0.026)	0.633 (0.040)	0.615 (0.027)	0.282 (0.071)	0.746 (0.061)	0.756 (0.029)
6	0.433 (0.027)	0.730 (0.015)	0.752 (0.006)	0.378 (0.014)	0.735 (0.020)	0.728 (0.021)	0.352 (0.077)	0.848 (0.025)	0.835 (0.020)

Table S7: Detailed performance of ucb on Enamine

Dataset	Enamine50k			EnamineHTS0.1			EnamineHTS0.2		
Arch	MLP	GNN	GraphMVP	MLP	GNN	GraphMVP	MLP	GNN	GraphMVP
1	0.011 (0.007)	0.011 (0.007)	0.011 (0.007)	0.002 (0.000)	0.002 (0.000)	0.002 (0.000)	0.003 (0.002)	0.003 (0.002)	0.003 (0.002)
2	0.074 (0.020)	0.159 (0.035)	0.171 (0.050)	0.037 (0.008)	0.060 (0.040)	0.060 (0.038)	0.070 (0.027)	0.143 (0.055)	0.137 (0.055)
3	0.158 (0.024)	0.375 (0.027)	0.391 (0.038)	0.074 (0.005)	0.282 (0.075)	0.276 (0.048)	0.133 (0.041)	0.483 (0.047)	0.470 (0.040)
4	0.248 (0.018)	0.555 (0.026)	0.576 (0.030)	0.159 (0.034)	0.500 (0.068)	0.474 (0.051)	0.219 (0.059)	0.714 (0.034)	0.696 (0.036)
5	0.343 (0.007)	0.660 (0.018)	0.686 (0.006)	0.256 (0.054)	0.625 (0.054)	0.629 (0.047)	0.284 (0.067)	0.823 (0.012)	0.816 (0.026)
6	0.411 (0.006)	0.734 (0.017)	0.757 (0.007)	0.344 (0.055)	0.715 (0.038)	0.720 (0.017)	0.362 (0.074)	0.869 (0.005)	0.876 (0.014)

Table S8: Detailed performance of GLARE on Enamine

Dataset	Enamine50k			EnamineHTS0.1			EnamineHTS0.2		
Arch	MLP	GNN	GraphMVP	MLP	GNN	GraphMVP	MLP	GNN	GraphMVP
1	0.011 (0.007)	0.011 (0.007)	0.011 (0.007)	0.002 (0.000)	0.002 (0.000)	0.002 (0.000)	0.003 (0.002)	0.003 (0.002)	0.003 (0.002)
2	0.070 (0.016)	0.158 (0.023)	0.162 (0.026)	0.033 (0.006)	0.121 (0.011)	0.094 (0.031)	0.053 (0.025)	0.165 (0.084)	0.201 (0.098)
3	0.151 (0.033)	0.385 (0.025)	0.378 (0.025)	0.076 (0.007)	0.340 (0.058)	0.297 (0.027)	0.124 (0.036)	0.472 (0.086)	0.464 (0.135)
4	0.235 (0.038)	0.558 (0.027)	0.576 (0.010)	0.174 (0.021)	0.542 (0.051)	0.506 (0.039)	0.256 (0.053)	0.697 (0.045)	0.712 (0.060)
5	0.322 (0.039)	0.670 (0.017)	0.680 (0.009)	0.291 (0.033)	0.657 (0.032)	0.660 (0.024)	0.376 (0.070)	0.804 (0.021)	0.805 (0.031)
6	0.412 (0.030)	0.746 (0.005)	0.746 (0.010)	0.377 (0.031)	0.734 (0.021)	0.732 (0.015)	0.469 (0.069)	0.868 (0.018)	0.862 (0.014)

1.3 Analysis on structural similarity threshold for activity cliff selection

To evaluate the impact of the Tanimoto similarity threshold (T_s) on our framework, we conducted a sensitivity analysis by varying the threshold value. Following the experimental designs established in previous studies, $T_s = 0.6$ was employed as the default setting for defining structural sensitivity and activity cliffs. To ensure that the performance of **ACActive** is not overly dependent on this specific parameter, we further evaluated the model using thresholds of $T_s = 0.4$ and $T_s = 0.8$.

As summarized in Table S9 and S10, the experimental results across both MLP and GraphMVP-based GNN architectures lead to the following observations:

Table S9: Detailed performance comparison of MLP and GraphMVP across different Tanimoto similarity thresholds. We evaluated with enrichment factor.

Arch	Metric	ALDH1		PKM2		VDR	
		Round 10	Round 16	Round 10	Round 16	Round 10	Round 16
MLP	Sim 0.4	5.247	**5.740**	4.630	5.966	6.504	8.170
	Sim 0.6	**5.339**	5.677	5.704	**6.090**	**6.583**	**8.525**
	Sim 0.8	5.119	5.586	**5.735**	6.025	5.552	8.229
GraphMVP	Sim 0.4	5.363	6.173	5.374	5.779	6.358	8.229
	Sim 0.6	**5.844**	**6.303**	**6.779**	**7.271**	**6.901**	8.407
	Sim 0.8	5.272	6.023	6.118	6.339	5.949	**8.466**

Table S10: Performance comparison of MLP and GraphMVP on EnamineHTS0.2 datasets across different Tanimoto similarity thresholds. We evaluated with retrieval rate.

Arch	Metric	Enamine50k		EnamineHTS0.1		EnamineHTS0.2	
		Round 4	Round 6	Round 4	Round 6	Round 4	Round 6
MLP	Sim 0.4	0.263	0.431	0.163	0.370	**0.274**	0.491
	Sim 0.6	**0.274**	**0.450**	**0.190**	**0.381**	0.264	0.484
	Sim 0.8	0.259	0.430	0.170	0.380	0.270	**0.494**
GraphMVP	Sim 0.4	0.558	0.753	0.509	0.763	0.704	0.884
	Sim 0.6	**0.581**	**0.760**	0.521	**0.779**	0.718	0.888
	Sim 0.8	0.563	0.752	**0.534**	0.773	**0.740**	**0.893**

1.4 Computational cost

The computational efficiency of the proposed method was evaluated to demonstrate its practical applicability to large-scale chemical libraries. Theoretically, calculating all possible pairwise relationships within a dataset of size n entails a worst-case complexity of $O(n^2)$. However, the proposed framework optimizes this process by restricting the core computations to identified activity cliff pairs. By prioritizing these structurally sensitive regions rather than performing exhaustive pairwise comparisons across the entire chemical space, the method maintains a manageable computational load.

The scalability was empirically validated across datasets of various sizes, ranging from ALDH1 ($\sim$100,000 molecules) to the EnamineHTS library ($\sim$2.1 million molecules). As detailed in the performance tables, the runtime was compared against the standard greedy acquisition strategy and the GLARE baseline.

Table S11: Computational cost analysis on ALDH1 dataset. Comparison of training and inference time (in seconds) across different methodologies and architectures.

Arch	Greedy		GLARE		ACActive	
	Train (Sec)	Inference (Sec)	Train (Sec)	Inference (Sec)	Train (Sec)	Inference (Sec)
MLP	3.584	1.103	13.932	1.435	4.900	1.408
GraphMVP	25.862	12.966	53.334	14.878	27.843	13.977

Table S12: Computational cost analysis on EnamineHTS0.2 dataset. Comparison of training and inference time (in seconds) across different methodologies and architectures.

Arch	Greedy		GLARE		ACActive	
	Train (Sec)	Inference (Sec)	Train (Sec)	Inference (Sec)	Train (Sec)	Inference (Sec)
MLP	6.900	41.600	35.694	65.036	14.707	50.149
GraphMVP	19.727	153.36	65.656	271.08	37.362	251.69

1.5 Analysis on hyperparameter sensitivity

A systematic hyperparameter search was conducted across a range of values, including $\alpha \in \{0.05, 0.1, 0.2\}$ and $\lambda \in \{0.001, 0.01, 0.1\}$, to assess the performance of the proposed method under different configurations.

As detailed in the sensitivity tables (e.g., Table S13), the results indicate that the performance remains relatively consistent across the tested hyperparameter settings. This suggests that the method is not highly sensitive to the specific choice of these parameters within the evaluated ranges. Based on these observations, intermediate values of $\alpha = 0.1$ and $\lambda = 0.01$ were employed as the representative settings for the main experiments.

The choice of a moderate value for α is based on the consideration of the trade-off between bioactivity and structural sensitivity. While incorporating the activity cliff score is intended to prioritize structurally sensitive molecules, an excessively large α may cause this component to dominate the acquisition function. This could potentially overshadow the influence of the predicted bioactivity, leading to a biased selection process. Therefore, $\alpha = 0.1$ was selected to ensure a balanced integration of both components, maintaining stable performance across different screening scenarios.

Table S13: Hyperparameter sensitivity analysis on EnamineHTS0.2 dataset across different values of Alpha and Lambda. We evaluated with retrieval rate.

Metric	Value	MLP		GNN		GraphMVP	
		Round 4	Round 6	Round 4	Round 6	Round 4	Round 6
	0.05	0.259	0.511	0.689	0.871	0.735	0.873
Alpha	0.1	0.264	0.484	0.722	0.880	0.718	0.888
	0.2	0.250	0.491	0.742	0.883	0.737	0.887
	0.001	0.273	0.470	0.700	0.886	0.711	0.881
Lambda	0.01	0.264	0.484	0.722	0.880	0.718	0.888
	0.1	0.259	0.511	0.714	0.868	0.718	0.901

Table S14: Hyperparameter sensitivity analysis on ALDH1 dataset across different values of Alpha and Lambda. We evaluated with enrichment factor.

Metric	Value	MLP		GNN		GraphMVP	
		Round 10	Round 16	Round 10	Round 16	Round 10	Round 16
	0.05	5.217	5.411	5.466	5.626	5.211	5.390
Alpha	0.1	5.339	5.677	5.436	5.960	5.844	6.303
	0.2	5.661	5.701	5.497	6.027	5.497	6.260
	0.001	5.010	5.646	5.430	6.165	5.357	5.957
Lambda	0.01	5.339	5.677	5.436	5.960	5.844	6.303
	0.1	5.065	5.795	5.308	5.661	5.333	5.992

1.6 Analysis on robustness

To evaluate the resilience of the proposed method against experimental errors, a robustness analysis was performed by introducing synthetic label noise. Specifically, 5% and 10% of the active compounds were randomly flipped to inactive to simulate potential mislabeling in bioassay data.

On the EnamineHTS dataset, the model demonstrated high stability, with performance remaining largely unaffected by the noise. Even at a 10% noise level, only a marginal decrease of approximately 2% was observed. This result indicates that the framework effectively distinguishes true structural sensitivity from stochastic noise, maintaining its predictive integrity in large-scale screening scenarios.

In contrast, the ALDH1 dataset exhibited a more pronounced performance degradation under noise. This susceptibility is likely attributed to the smaller scale of the dataset, where label perturbations have a disproportionately larger impact on the model's decision boundary. However, as summarized in Table S16, the proposed method consistently outperformed baseline approaches under identical noise conditions. This confirms the relative robustness of the framework, demonstrating its superior ability to prioritize high-confidence structural signals even in data-constrained and noisy environments.

Table S15: Robustness analysis on Enamine dataset under synthetic label noise (0%, 5%, and 10%). We evaluated with retrieval rate.

Metric	Level	MLP		GNN		GraphMVP	
		Round 4	Round 6	Round 4	Round 6	Round 4	Round 6
Noise	0%	0.264	0.484	0.722	0.880	0.718	0.888
	5%	0.250	0.475	0.719	0.884	0.726	0.883
	10%	0.247	0.474	0.686	0.870	0.664	0.872

Table S16: Robustness analysis on ALDH1 dataset comparing ACActive and GLARE under synthetic label noise (0%, 5%, and 10%). We evaluated with enrichment factor.

Method	Noise	MLP		GNN		GraphMVP	
		Round 10	Round 16	Round 10	Round 16	Round 10	Round 16
ACActive	0%	5.339	5.677	5.436	5.960	5.844	6.303
	5%	4.578	5.441	5.126	5.559	5.211	5.649
	10%	3.811	4.398	4.986	4.949	4.547	4.571
GLARE	0%	4.992	5.283	5.217	5.252	5.515	5.779
	5%	4.967	5.185	4.517	4.925	4.912	5.287
	10%	3.811	3.984	4.072	4.630	3.482	4.197

1.7 Additional ablation study

To isolate the effect of cliff-aware training, an ablation study was conducted in which the backbone model was trained independently without incorporating the cliff-aware objective. In this setup, the cliff scoring module was trained separately and utilized solely for the acquisition function.

This experimental design ensures that cliff-related information is not used to guide the representation learning of the backbone model, while still enabling the use of cliff-based acquisition. By establishing this baseline, we disentangle the individual contribution of cliff-aware training from that of the acquisition strategy.

The results demonstrate that incorporating cliff-aware training provides additional performance gains beyond those achieved by the acquisition strategy alone, confirming the synergistic effect of our proposed framework.

Table S17: Ablation study of ACActive across different architectures on ALDH1, PKM2, and VDR datasets. We evaluated with enrichment factor.

Arch	Method	ALDH1		PKM2		VDR	
		Round 10	Round 16	Round 10	Round 16	Round 10	Round 16
MLP	Ablation	5.174	5.685	6.118	5.655	6.425	8.111
	ACActive	5.339	5.677	5.704	6.090	6.583	8.525
GNN	Ablation	5.040	5.953	3.472	5.469	3.490	4.203
	ACActive	5.436	5.960	5.456	6.339	6.346	8.229
GraphMVP	Ablation	5.552	5.960	6.200	6.960	5.949	7.518
	ACActive	5.844	6.303	6.779	7.271	6.901	8.407

1.8 Statistical analysis

To ensure the statistical rigor of our findings, particularly in large-scale datasets where performance margins may be narrow, we conducted a detailed analysis to distinguish algorithmic improvement from run-to-run variance.

The reliability of the proposed method was validated by reporting results from five independent runs ($n = 5$) using different random initializations. As summarized in the detailed performance reports, our framework consistently outperformed the baseline models across the individual seeds. This observation of variance across initializations confirms that the reported gains are not artifacts of specific seeds but represent a robust improvement in model performance.

Furthermore, the performance enhancement was consistently observed across multiple datasets with varying chemical properties and hit densities. The simultaneous improvements across diverse datasets and across all five random seeds provide strong empirical evidence that the observed performance gains are statistically significant and not attributable to chance. This consistency across independent experiments reinforces the generalizability of the proposed framework in various drug discovery scenarios.

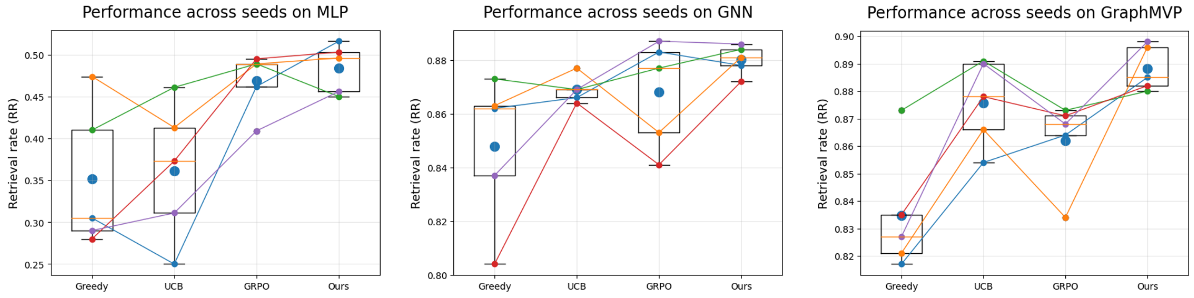

Figure S1: Comparison between baselines and our model on the EnamineHTS0.2 dataset, showing both the mean and individual results for each seed.

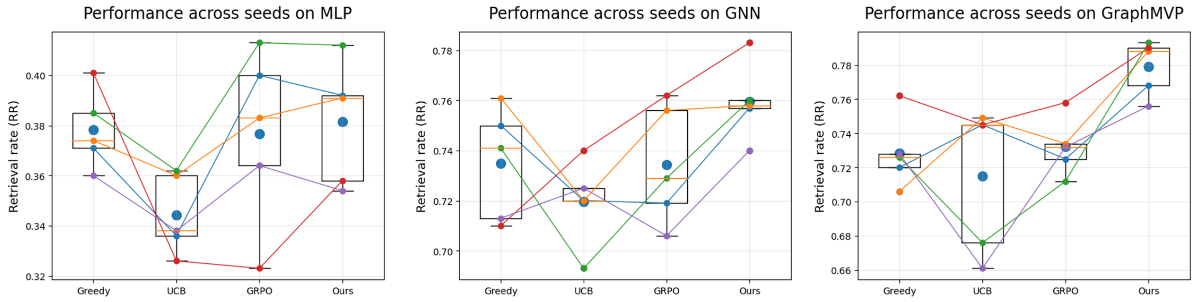

Figure S2: Comparison between baselines and our model on the EnamineHTS0.1 dataset, showing both the mean and individual results for each seed.

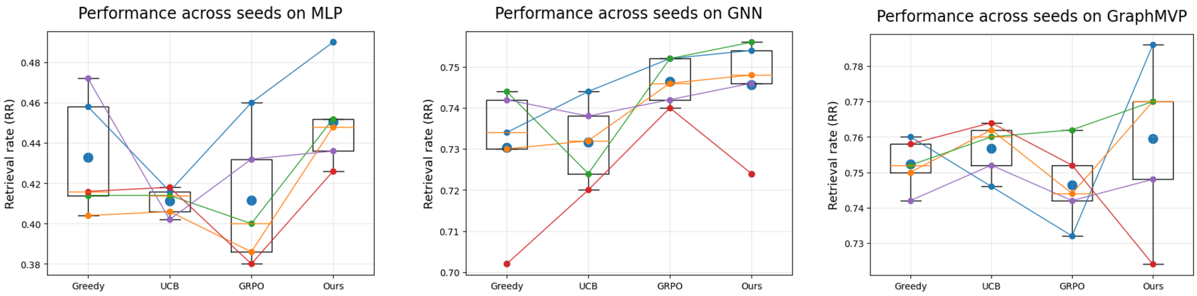

Figure S3: Comparison between baselines and our model on the Enamine50k dataset, showing both the mean and individual results for each seed.

2 Supplementary Methods

2.1 Comparison model settings

2.1.1 Baseline acquisition strategy

Greedy: Selects unlabeled samples with the highest predicted probability of being active according to the current model, i.e., $c(x) = \Pr(y = 1 \mid x)$. This strategy exploits the model's current belief without explicitly accounting for uncertainty [1, 2].
Mutual Information: Selects samples that minimize the mutual information between model predictions and parameter. In practice, this is approximated using Bayesian neural networks to estimate predictive entropy and expected entropy [1].

$$a_i = \arg\min_n \big(H(y \mid x) - \mathbb{E}_M\big[H(y \mid x, \theta)\big]\big)\,. \tag{1}$$

UCB: Upper Confidence Bound selects samples by balancing exploitation and exploration using the mean and uncertainty of predictions, $c(x) = \mu(x) + \beta\sigma(x)$, where $\mu(x)$ and $\sigma(x)$ denote the predictive mean and standard deviation, respectively.
GLARE: A reinforcement learning-based active learning method that learns an acquisition policy via GRPO instead of using predefined heuristics [3]. We used the hyperparameter settings reported in [3].

2.1.2 Backbone model

MLP: A multilayer perceptron trained on fixed molecular fingerprints (ECFP[5]), serving as a simple non-graph baseline for molecular activity prediction [6]. We used ECFP as feature for MLP input.
GNN: A graph neural network that directly models molecular graphs by aggregating atom-level features, capturing local structural information through message passing. We used GIN encoder [7].
GraphMVP: A pretrained graph neural network that leverages large-scale molecular pretraining with multi-view objectives, providing rich and transferable molecular representations for downstream prediction tasks [4].

2.2 Implementation details

Models are trained using the Adam optimizer with a learning rate of 3e-4 and a batch size of 64. For MLP and GIN, we used a 1,024-dimensional hidden layer and we used 128-dimensional hidden layer for cliff scoring module. The number of training epochs is set to 50 following previous works [1, 3]. The overall training objective is a weighted sum of the backbone classification loss and the activity cliff prediction loss. λ is set to 1e-2.

References

[1] Derek van Tilborg and Francesca Grisoni. Traversing chemical space with active deep learning for low-data drug discovery. *Nature Computational Science*, 4(10):786–796, 2024.

[2] David E Graff, Eugene I Shakhnovich, and Connor W Coley. Accelerating high-throughput virtual screening through molecular pool-based active learning. *Chemical Science*, 12(22):7866–7881, 2021.

[3] Yicong Chen, Jiahua Rao, Jiancong Xie, Dahao Xu, Zhen Wang, and Yuedong Yang. Reinforced active learning for large-scale virtual screening with learnable policy model. In *Proceedings of the 39th International Conference on Neural Information Processing Systems*, 2025.

[4] Liu, Shengchao and Wang, Hanchen and Liu, Weiyang and Lasenby, Joan and Guo, Hongyu and Tang, Jian. Pre-training Molecular Graph Representation with 3D Geometry. *Proceedings of the Thirteenth International Conference on Learning Representations*, 2022.

[5] David Rogers and Mathew Hahn. Extended-connectivity fingerprints. *Journal of Chemical Information and Modeling*, 50(5):742–754, 2010.

[6] David E Rumelhart, Geoffrey E Hinton, and Ronald J Williams. Learning representations by back-propagating errors. *Nature*, 323(6088):533–536, 1986.

[7] Keyulu Xu, Weihua Hu, Jure Leskovec, and Stefanie Jegelka. How powerful are graph neural networks? *Advances in Neural Information Processing Systems*, 31, 2019.
